# Inhibition of NF-κB Signaling Alters Acute Myelogenous Leukemia Cell Transcriptomics

**DOI:** 10.3390/cells9071677

**Published:** 2020-07-12

**Authors:** Håkon Reikvam

**Affiliations:** 1 Institute of Clinical Science, University of Bergen, 5020 Bergen, Norway; Hakon.Reikvam@med.uib.no; 2 Department of Medicine, Haukeland University Hospital, 5021 Bergen, Norway

**Keywords:** acute myelogenous leukemia, nuclear factor-κB, transcriptomic, cytokines, metabolism, immune system, intracellular signaling

## Abstract

Acute myelogenous leukemia (AML) is an aggressive hematological malignancy. The pathophysiology of the disease depends on cytogenetic abnormalities, gene mutations, aberrant gene expressions, and altered epigenetic regulation. Although new pharmacological agents have emerged during the last years, the prognosis is still dismal and new therapeutic strategies are needed. The transcription factor nuclear factor-κB (NF-κB) is regarded a possible therapeutic target. In this study, we investigated the alterations in the global gene expression profile (GEP) in primary AML cells derived from 16 consecutive patients after exposure to the NF-κB inhibitor BMS-345541. We identified a profound and highly discriminative transcriptomic profile associated with NF-κB inhibition. Bioinformatical analyses identified cytokine/interleukin signaling, metabolic regulation, and nucleic acid binding/transcription among the major biological functions influenced by NF-κB inhibition. Furthermore, several key genes involved in leukemogenesis, among them *RUNX1* and *CEBPA*, in addition to *NFKB1* itself, were influenced by NF-κB inhibition. Finally, we identified a significant impact of NF-κB inhibition on the expression of genes included in a leukemic stem cell (LSC) signature, indicating possible targeting of LSCs. We conclude that NF-κB inhibition significantly altered the expression of genes central to the leukemic process.

## 1. Introduction

Acute myelogenous leukemia (AML) is a heterogeneous and aggressive malignancy characterized by bone marrow accumulation of immature leukemic blasts that suppress normal hematopoiesis [1]. The median age at the time of diagnosis is 65–70 years [1], and for younger patients receiving the most intensive therapy, the median long-term AML-free survival is 45–50% [2]. However, older and unfit patients with severe comorbidities cannot receive the most intensive treatment, i.e., highly cytotoxic chemotherapy or allogeneic hematopoietic stem cell transplantation (allo-HSCT), due to an unacceptable risk of treatment-related mortality [3]. The average survival for those patients receiving supportive therapy alone is only 2–4 months, and very few survive longer than one year [3]. All these observations clearly illustrate the need for new and more effective therapeutic approaches with acceptable toxicity, especially for elderly patients. 

Abnormal constitutive activation of intracellular signal transduction could enhance the survival and proliferation of AML cells and could occur due to genetic abnormalities [4,5]. Several signaling cascades finally lead to the activation of the transcription factor nuclear factor-κB (NF-κB) [6]. The NF-κB family of transcription factors was identified more than 30 years ago as a binding interactor for the κ light chain enhancer in B-lymphocytes [7]. This complex is activated by several stimuli, including pro-inflammatory cytokines, mitogens, growth factors, and stress-inducing agents [8,9,10]. NF-κB is involved in the regulation of multiple fundamental cellular processes, including cell proliferation, cell survival, intercellular communication, stress responses, regulation of angiogenesis as well as inflammation, and even carcinogenesis/leukemogenesis [8,9,10].

Constitutive NF-κB activation has been reported both in patient studies as well as in vitro and experimental in vivo models of AML [6], and NF-κB seems to of particular importance for the subset of CD34^+^ AML cells [11]. These CD34^+^ leukemic cells are believed to be the main compartment for leukemic stem cells (LSCs) for most patients, a leukemic cell subset regarded to be important for chemoresistance and, ultimately, AML relapse [12,13,14]. Furthermore, several studies suggest a crucial role of NF-κB in leukemogenesis also through indirect effects mediated by bi-directional crosstalk between the leukemic cells and their neighboring stromal cells [15,16].

Given the central role of NF-κB in cancer biology, this has also increased the interest for pharmacological targeting of this transcription factor in cancer therapy [9]. Several NF-κB inhibitors have been identified [17]. Some of them have entered preclinical and clinical trials [9], including the pharmacological agent BMS-345541, which selectively interacts with inhibitors of κB (IκB) kinase (IKK). The inhibition of IKK leads to the decreased phosphorylation of serine residues of IκB with the subsequent ubiquitination and proteolysis of IκB, and thereby, inhibition of NF-κB-mediated gene transcription. BMS-345541 is regarded as a highly selective inhibitor of NF-κB function, and previous studies have shown a high degree of NF-κB inhibition by BMS-345541 [18,19,20]. 

The aim of the present study was to investigate the gene expression profile (GEP) associated with the pharmacological intervention by BMS-345541 in primary human AML cells. Furthermore, we wanted to identify the major biological systems and signaling pathways that were altered by this NF-κB inhibitor in primary human AML cells.

## 2. Materials and Methods

### 2.1. AML Patient Population and Preparation of Primary AML Cells

The study was approved by the local Ethics Committee (REK III 2013/634, University of Bergen, Norway) and samples were collected after written informed consent. Our study included 16 consecutive and thereby unselected patients (11 males and five females; median age 70 years with range 39–87 years), with high relative and absolute peripheral blood blast counts (>5 × 10^9^/L) (Table 1). All patients were diagnosed according to the World Health Organization (WHO) criteria [21], and most of them had de novo disease (Table 1). All AML cell samples were obtained from peripheral blood, the leukemic cells were isolated by density gradient separation (Lymphoprep, Axis-Shield, Oslo, Norway), and gradient-separated cells contained at least 95% leukemic blasts. Cells were stored frozen in liquid nitrogen until used in the experiments [22].

### 2.2. In Vitro Culture of Human AML Cells Before Analysis of Gene Expression

AML blasts (2 × 10^6^ cells in 2 mL) were cultured in 24 well culture plates (Costar 3524; Cambridge, MA, USA) for 48 h in StemSpan SFEM™ medium (referred to as StemSpan; Stem Cell Technologies; Vancouver, BC, Canada), supplemented with 100 µg/mL of gentamicin. The NF-κB inhibitor BMS-345541 was purchased from Sigma-Aldrich Corp. (St. Louis, MO, US). Stock solutions of BMS-345541 were prepared in dimethylsulphoxide (DMSO), and the final DMSO concentration in our inhibitor-free control cultures was the same as in the BMS-345541 containing cultures. The final concentration used for BMS-345541 was 10 µM; this concentration has also been used in previous studies of in vitro cultured primary AML cells and has been shown to inhibit the constitutive cytokine release [16,23,24]. The final DMSO concentration did not have any effect on the in vitro proliferation or viability of cryopreserved primary human AML cells [16,23].

### 2.3. RNA Preparation, Labelling and Microarray Hybridisation

All microarray experiments were performed using the Illumina iScan Reader, which is based on fluorescence detection of biotin-labeled cRNA [25]. Total RNA (300 ng from each sample) was reversely transcribed, amplified, and Biotin-16-UTP-labeled using the Illumina TotalPrep RNA Amplification Kit (Applied Biosystems/Ambion, USA). The amount and quality of the B biotin-labelled cRNA were controlled both by the NanoDrop spectrophotometer and by the Agilent 2100 Bioanalyzer before 750 ng of the biotin-labeled cRNA was hybridized to the HumanHT-12 V4 Expression BeadChip according to manufacturer’s instructions. The HumanHT-12 V4 BeadChip targets 47,231 probes derived mainly from genes in the NCBI RefSeq database (Release 38).

### 2.4. Statistical and Bioinformatical Analyses 

The data from the scanning of arrays on the IlluminaScan Reader were investigated in GenomeStudio (Illumina) and J-Express 2012 (MolMine AS, Bergen, Norway) for quality control measures [26]. All arrays within each experiment were quintile normalized to be comparable before being compiled into an expression profile data matrix. The Geneontology (http://geneontology.org/) database was used to identify ontology annotations. In the analysis of the functional network, we used the STRING (version 11.0) [27], and the PANTHER databases (version 14.0) to search for gene ontology profiles [28]

## 3. Results

### 3.1. NF-κB Inhibition Alters the Global GEP of Primary Human AML Cells

We first investigated the alterations in the global GEP for primary AML cells derived from the 16 patients; the cells were cultured with and without the NF-κB inhibitor BMS-345541. The global profiles for BMS-345541-exposed cells were compared with the GEP for cells from the corresponding inhibitor-free control cultures for each of the 16 patients. We performed a feature subset selection (FSS) analysis, and based on this analysis, we were able to discriminate between genes upregulated and downregulated in the BMS-345541-containing cultures. A total of 277 genes showed a fold change greater than ±3 and a t-score of at least ±2.5, and these 277 genes were regarded to be highly discriminative between cells cultured with either BMS-345541 or medium alone (Table S1). Among the 277 genes, 183 genes were downregulated, and 94 were upregulated. 

**Figure 1:**
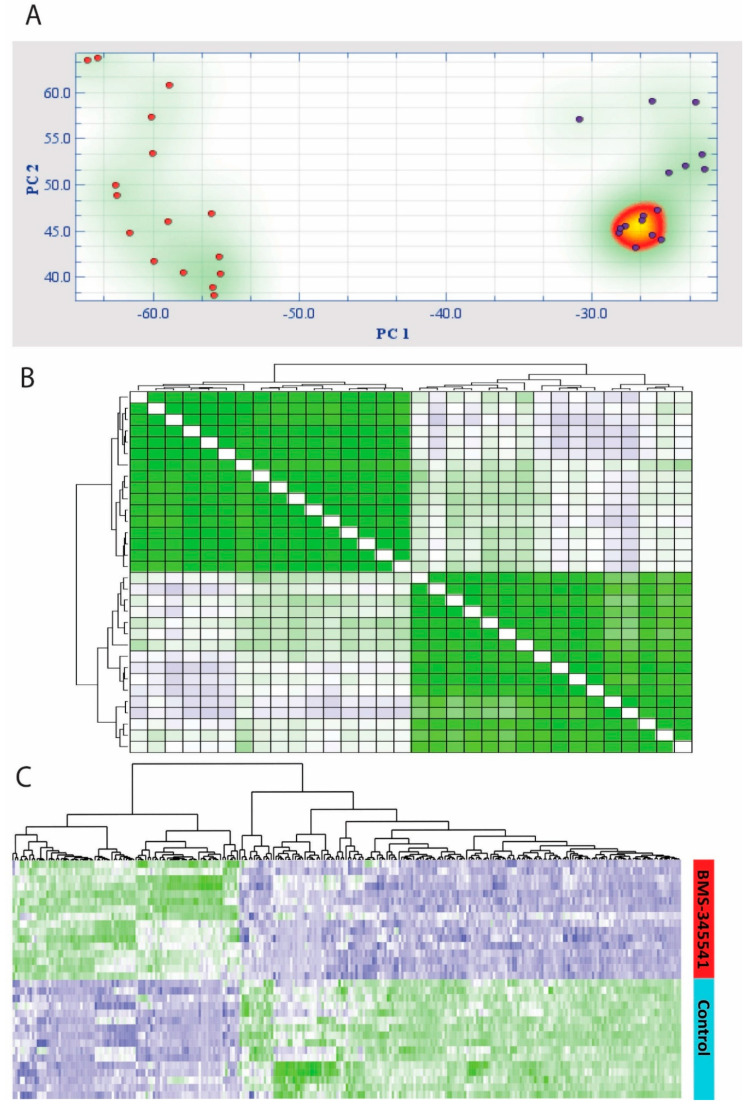
Effects of nuclear factor-κB (NF-κB) inhibition on the global gene expression profile of primary human acute myelogenous leukemia (AML) cells: We performed a feature subset selection (FSS) analysis and identified 277 genes that were differently expressed (i.e., fold change at least ±3, t-score at least ±2.5) when comparing cultures treated with the NF-κB inhibitor BMS-345541 and the corresponding control cultures. Most of these genes (183/277) were downregulated, and only 94 were upregulated in cells exposed to the NF-κB inhibitor. (**A**) By performing a principal component analysis (PCA), we identified the first principal component to be highly discriminative for cells exposed to the NF-κB inhibitor compared with control cells. (**B**) Discriminative hierarchical clustering with the distance matrix. The correlation visualization matrix displays the pairwise correlation between the 32 different cell cultures. Green and blue colors indicate high positive or negative correlation, respectively, between samples. (**C**) A hierarchical cluster analysis based on the 277 differentially expressed genes was finally performed (Pearson’s correlation, complete linkage). Green color indicates upregulated genes, and blue color indicates downregulated genes in the BMS-345541 cultures compared with controls. The red bar to the right in the figure indicates intervention cultures, whereas purple indicates control cultures. The hierarchical cluster analysis clearly identified two patient/cell clusters that corresponded to the BMS-345541 containing and control cultures and two gene expression clusters that corresponded to up- and downregulated genes.

We performed a principal component analysis (PCA) based on the 277 highly discriminative genes; the first principal component was highly discriminative for cells in the intervention cultures compared to cells in control cultures (Figure 1A). This observation was further supported by the highly discriminative hierarchical clustering with distance matrix analysis (Figure 1B), indicating a high degree of correlation between cells in intervention cultures versus control cultures. A hierarchical cluster analysis was finally performed (Pearson’s correlation, complete linkage; Figure 1C. This last analysis showed that the upregulated and downregulated genes in the two distinct clusters were highly consistent through the two groups, and the obtained profile could be used to highly discriminate the intervention and control cell cultures.

### 3.2. Overrepresentation Analysis Shows That Genes Involved in Metabolism and Immune Regulation Are Downregulated by NF-κB Inhibition 

**Figure 2:**
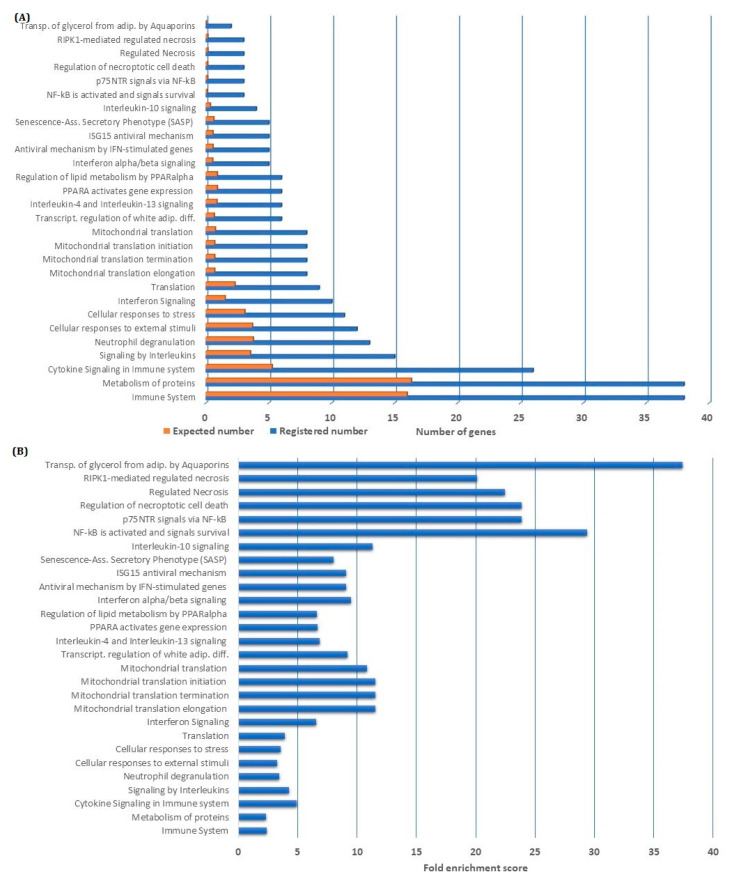
Gene ontology GO enrichment analysis of genes being downregulated by nuclear factor-κB (NF-κB) inhibition: We performed a GO enrichment analysis based on the 183 genes that were downregulated by NF-κB inhibition; the terms Biological Process and Reactome Pathways were used for annotation. The GO-terms identified by a false discovery rate (FDR) <0.05 are presented in the figure. (**A**) For each of the identified GO-terms, we present the number of identified genes (blue column) and the number of genes that would be expected to be identified by coincidence (orange column). (**B**) The fold enrichment score, i.e., representing the degree to which the identified downregulated genes are over-represented in the terms, is also presented for the identified GO-terms.

We further investigated the class of genes and their annotations to identify specific pathways affected by the downregulated genes. We then performed an overrepresentation analysis based on all 183 highly discriminative genes that were downregulated by NF-κB inhibition. Using the Geneontology database supported by the Panther database, we were able to perform a gene ontology (GO) enrichment analysis. By using the term Biological process, we investigated the overrepresentation of GO-terms among the 183 genes (Table S1), and by exploring the Reactome pathways, we identified several GO-terms enriched among the genes downregulated by NF-κB inhibition (Figure 2). We analyzed both terms for overrepresentation of genes, i.e., the number of genes among the downregulated genes compared with the number of genes expected to be found by coincidence, and in addition, we investigated the fold enrichment score. Some of the highly enriched GO-terms reflect biologic processes known to be involved in leukemogenesis and include immune system/signaling, protein metabolism, interleukin signaling, translation and interferon signaling together with NF-κB activation itself (Figure 2).

### 3.3. Genes Encoding Proteins That Are Crucial for Fundamental Cellular Functions Are Influenced by NF-κB Inhibition 

**Figure 3:**
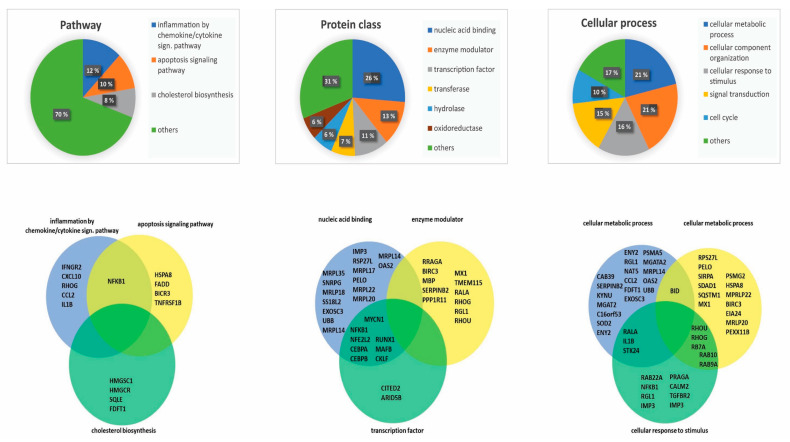
Classification of downregulated genes by BMS-345541 inhibition: We classified the 183 genes downregulated by nuclear factor-κB (NF-κB) inhibition according to their biological function by using the PANTHER database. As indicated at the top of the figure, we selected the three major terms pathway (**left**), protein class (**middle**), and cellular process (**right**) for detailed analyses. We were then able to identify major subgroups within each of these three main terms (upper part of the figure), as indicated by the corresponding diagrams in the lower part of the figure. For each of the three main terms (i.e., pathway/protein class/cellular process), we then selected the three largest subgroups of downregulated genes (see the lower part). For each main term, the three selected subgroups/terms are presented as partly overlapping circles, and the individual genes included in the various subgroups are indicated.

We analyzed the major GO terms associated with the genes that were downregulated by NF-κB inhibition. We used the PANTHER database to classify proteins encoded by the 183 downregulated genes [28]. We then searched for functional classification analysis and used the three major terms (i) Pathway, (ii) Protein class, and (iii) Cellular process (Figure 3). In each of these classes, we further investigated the three largest subcategories/terms. In the setting of pathway analysis, we identified inflammation mediated by the chemokine/cytokine signaling pathway, the apoptosis signaling pathway, and cholesterol biosynthesis to be the three major pathways (Figure 3 left). By investigating the single proteins of these three pathways, we identified several proteins encoded by downregulated genes involved in leukemogenesis, including NFKB1, CXCL10, and IL1B [5,6,29]. In the setting of protein class analysis, we identified nucleic acid binding, enzyme modulators, and transcription factors to be the three largest terms (Figure 3 middle). By investigating single encoding genes of these protein classes, we identified several proteins known to be involved in leukemogenesis, including NFKB1, RUNX1, and CEBPA [6,30,31]. Finally, for the term cellular process, we identified cellular metabolic process, cellular component organization, and cellular response to stimulus as the three major terms (Figure 3 right). By investigating single proteins included in these terms, we identified several proteins previously linked to leukemogenesis, including SOD2, BID, and RALA [32,33].

### 3.4. Network Analysis Identified Several Protein Networks to be Central in NF-κB Inhibition

**Figure 4:**
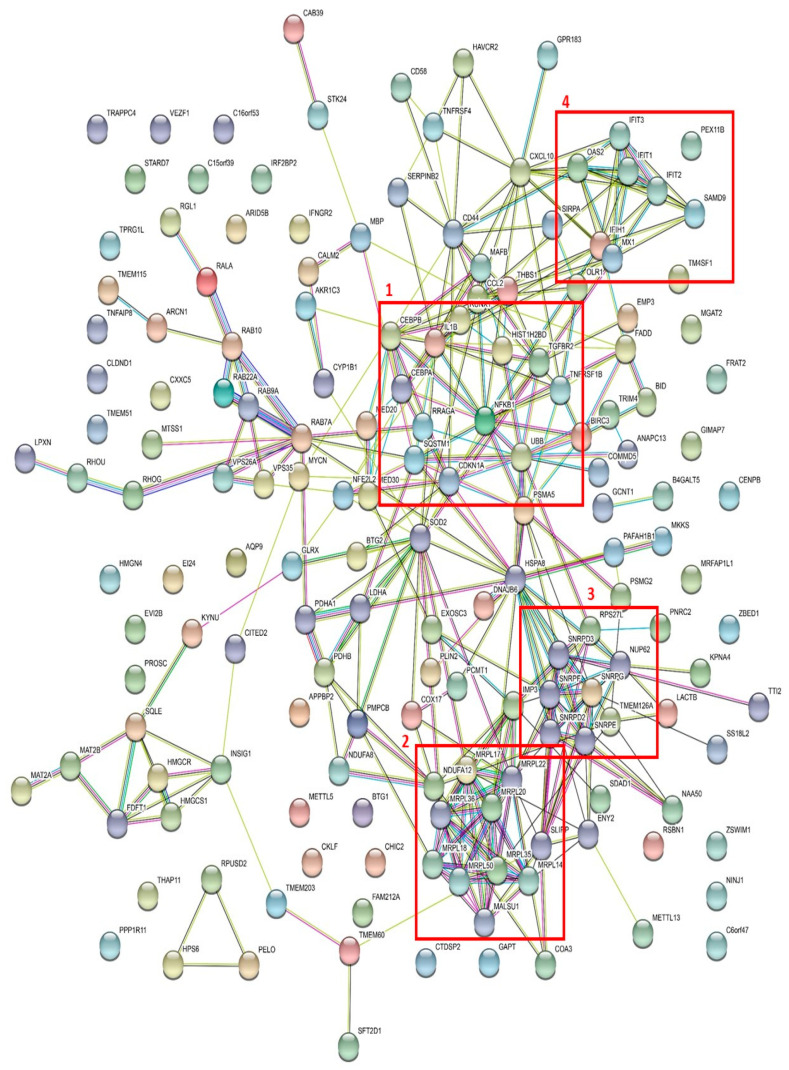
Network analysis of genes with downregulated expression after NF-κB inhibition: The figure presents a network analysis based on the proteins encoded by the 183 genes that were downregulated by NF-κB inhibition. By using the STRING database, we were able to identify a strong network where each individual node represents a single, protein-coding gene, and the number of connections indicates the number of specific and functionally relevant molecular interactions (i.e., functional interactions but not necessarily a direct binding between the molecules). Four extensive networks were identified by this analysis, as indicated in the Figure. 1, an nuclear factor-κB 1 (NFKB1) network; 2, a network including several mitochondrial ribosomal protein large (MRPL) proteins; 3, a network including several small nuclear ribonucleoprotein polypeptides (SNRP) molecules; and 4, a network including many interferon-induced protein with tetratricopeptide repeats (IFIT)

To further investigate whether the identified differences in GEP could be translated to a molecular network profile, we searched for interactions between the proteins encoded by the significantly downregulated genes in BMS-345541 exposed AML cells. We then used the STRING database [27]. Based on these downregulated genes, we identified a network based on a protein–protein interaction (PPI) enrichment *p*-value < 0.0005, i.e., the identified proteins show significantly more interactions than would be expected by coincidence alone. The results from this network analysis based on the downregulated genes are presented in Figure 4, and we identified four main interaction subsets. The main characteristics of the encoded proteins for each of the four networks are summarized in Table S2:

*Leukemogenesis-chemosensitivity.* This network included 10 proteins, and previous studies have shown that all the encoded proteins are involved in leukemogenesis and chemosensitivity in human AML (Table S2). This network included NFKB1 that is the DNA binding subunit of the NF-κB protein complex, and thereby, is essential for the transcriptional regulation by NF-κB [34]. 

*Mitochondrial proteins.* The second network of genes encoding several mitochondrial ribosomal protein large (MRPL) was also identified by this analysis of downregulated genes. Seven of the eight genes encoded proteins involved in mitochondrial protein synthesis/ribosomes, and mitochondrial markers, including different MRPLs, which have recently been linked to carcinogenesis [35,36]. The last gene encoded a mitochondrial protein involved in energy metabolism.

*Spliceosomal proteins/RNA.* The third network mainly including small nuclear ribonucleoprotein polypeptides (SNRP) was identified. Their biological functions are not fully understood, although they are believed to be important for the spliceosome [37]. 

*Interferon (IFN)-induced proteins.* The interferon-induced protein with tetratricopeptide repeats (IFITs) constitutes an important network that probably interacts with the transcription factors RUNX1, CEBPB, and CEBPA. IFITs are prominent interferon-stimulated genes, with critical molecular, biological, and oncogenic functions, and they are possibly important in the development and maintenance of malignant diseases [38].

To conclude, our interaction analysis of genes downregulated by NF-κB inhibition identified four main interaction networks, and previous studies suggest that all four downregulated networks included several genes encoding proteins that were important for leukemogenesis and chemosensitivity in human AML.

### 3.5. Genes Upregulated by NF-κB Inhibition Constitute a Less Pronounced Profile Compared to Downregulated Genes

Only 94 of the 277 differentially expressed genes were upregulated after exposure to the NF-κB inhibitor. Furthermore, the upregulated genes showed relatively few interactions, and no annotations were significant by the GO enrichment analysis. However, by using the STRING database, we were able to identify three minor networks of upregulated genes (Figure S1). The functional characteristics of the encoded proteins by these three networks are briefly described in Table S3, including the two proteins IGFBP7 and NR4A2 that have recently been linked to AML leukemogenesis [39,40].

### 3.6. The Leukemic Stem Cell GEP Signature Was Significantly Altered by NF-κB Inhibition

**Figure 5:**
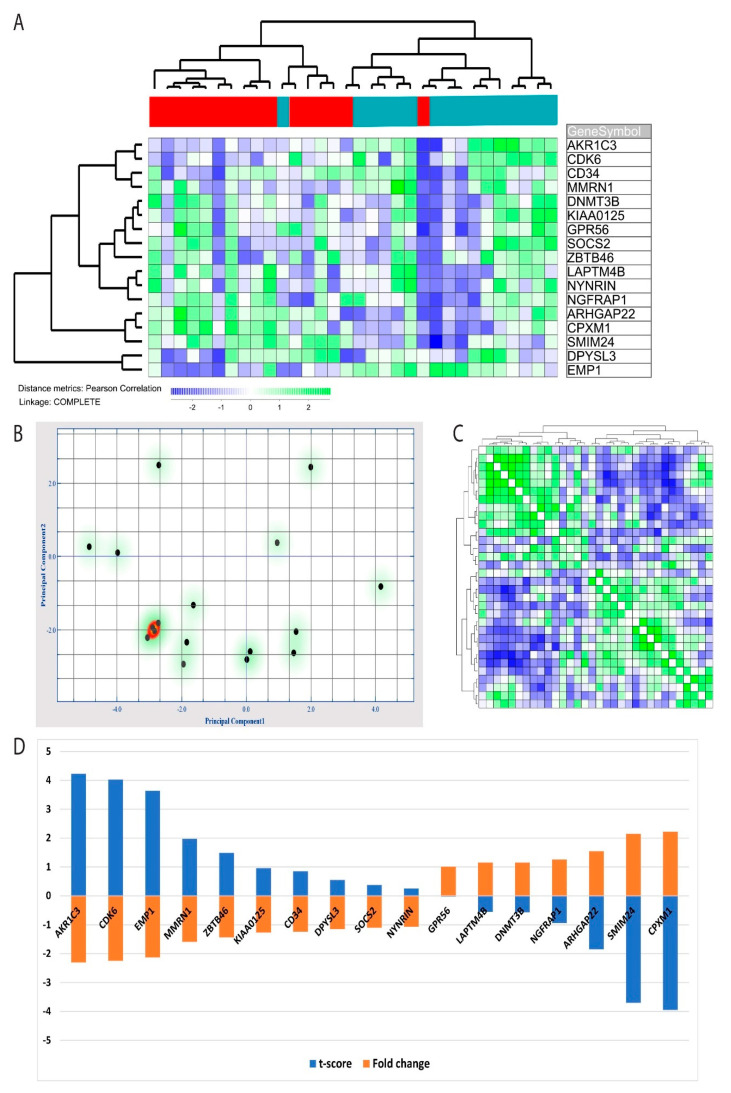
Alteration in acute myelogenous leukemia (AML) leukemia stem cell (LSC) gene expression profile (GEP) AML LSC GEP by nuclear factor-κB (NF-κB) inhibition: An LSC gene expression profile, including 17 genes has been identified in a previous study [14], and we, therefore, compared these 17 gene profiles in primary AML cells incubated with and without the BMS-345541 inhibitor. (**A**) Hierarchical clustering analysis was performed (Pearson’s correlation, complete linkage) based on the 17 genes. The green color indicates upregulated genes in the BMS-345541 containing cultures compared with the corresponding control cultures, whereas the blue color indicates downregulation. Red bars at the top of the figure indicate BMS-345541-containing cultures, and the purple bars indicate control cultures. The hierarchical cluster analysis identified two patient cell clusters corresponding to cells with/without BMS-345541, except for two outliers. (**B**) Principal component analysis based on the same 17 genes included in the LSC gene profile showed that the first and second principal components could explain 32.1% and 15.8% of the variance, respectively. (**C**) Discriminative hierarchical clustering with the distance matrix analysis indicated a high degree of correlation between cells from control cultures and the corresponding BMS-345541-containing cultures. (**D)** ANOVA analysis based on the 17 identified LSC-associated genes identified the t-score and fold change for each of these 17 genes, and these observations are present in the diagram.

NF-κB has previously been linked to LSCs in AML models [11], and we, therefore, investigated the alterations in a previously identified LSC GEP [14]. These authors identified a 17 gene LSC score based on identified LSC cell fractions, and this score could also be used for prognostication of AML patients [14]. We identified these 17 genes in our microarray data set. *AKR1C3* was the only of these 17 genes that was included among the 277 genes that were downregulated after NF-κB inhibition (Table S1). However, we found that the expression profile based only on these 17 genes was consistently altered by NF-κB inhibition, 10 of these genes were then downregulated, and seven genes were upregulated. By performing a hierarchical clustering based on these 17 genes, we found the profile to be highly discriminative between patient cells from control cultures and patient cells exposed to the BMS-345541 inhibitor; there was only one outlier in each group (Figure 5A). These observations were further supported by a principal component analysis based on the LSC gene expression profile, indicating that principal components 1 and 2 were able to identify 32.1% and 15.8% of the variance, respectively (Figure 5B). Furthermore, a hierarchical clustering analysis with distance matrix also illustrated how the 17 gene LSC profiles could discriminate between cells cultured with and without BMS-345541 (Figure 5C). Finally, by performing an ANOVA based on the 17 identified LSC genes, we could identify the t-score and fold change for each individual LSC profile gene, and these results are present in Figure 5D.

## 4. Discussion

Aberrant NF-κB signaling associated with gene transcription modulation is involved in the initiation, maintenance, and progression of several malignant diseases and are also common in hematological malignancies, including AML [9]. Hence, pharmacological inhibition of NF-κB is regarded as a possible therapeutic approach both as monotherapy and in combination with conventional chemotherapeutic agents. However, a better understanding of how such pharmacological targeting affects primary AML cells is needed. In our present study, we investigated primary AML cells derived from 16 consecutive AML patients. Our selection of patients reflects the expected heterogeneity of AML with regard to etiology, i.e., secondary or de novo, and biology of the disease, including cytogenetic and molecular genetic abnormalities [2]. Based on the culture of the primary AML cells under highly standardized in vitro conditions, we were able to compare GEP obtained from inhibitor-free control cultures with cells cultured in the presence of the NF-κB inhibitor BMS-345541. GEP in AML is a well-established methodological strategy with clinical relevance, it reflects cytogenetic and molecular genetic subclassifications, and has a prognostic impact [41,42]. Altered GEP during interventions could be an important tool for understanding the effect on biological systems. In the present study, we discovered a significant alteration in the transcriptomic profile after NF-κB intervention; 277 genes with significantly altered expression were identified, and these genes were highly discriminative for the intervention versus the control cells. Given the central role of NF-κB in the regulation of transcription, it is not surprising that the alteration in the transcriptomic profile is significant. These observations are also consistent with findings in other malignancies showing that NF-κB inhibition could significantly alter the transcriptomic profile [43].

AML is a very heterogeneous disease [1,4], this heterogeneity includes transcriptional regulation [41,44]. Leukemic cells derived from different patients, may therefore differ regarding the effect of NF-κB inhibition. We investigated only 16 patients, and this is too few patients to characterize differences between AML patients regarding the effect of NF-κB inhibition based on GEP of their leukemic cells. Despite the patient heterogeneity, we detected significant effects of NF-κB inhibition on AML cell gene expression profiles. The effects described in the present study should, therefore, be regarded as effects of NF-κB inhibition that are common for all or most AML patients across their heterogeneous biological characteristics. We would emphasize that additional studies are needed for a more detailed characterization of pharmacological effects of NF-κB inhibition that may be important for subsets of patients or single patients. 

We used the HumanHT-12 V4 Expression BeadChip technology for analysis of global gene expression profiles. Methodological approaches based on sequencing technology are now available and represent an alternative methodological strategy. However, in our opinion, the chip methodology used in our present study article also represents a highly standardized and reproducible methodological approach and for these reasons represent an acceptable methodological strategy.

We investigated the NF-κB inhibitor BMS-345541. This is a well-characterized inhibitor, and the mechanisms behind its inhibitory effect have been characterized in previous molecular studies, including Western blot studies of several cancer cell lines [17,18,45]. By using this well-characterized inhibitor, the risk of off-target effects is reduced. Furthermore, this inhibitor, as well as the proteasome inhibitor bortezomib, another drug with NF-κB inhibitory activity, could reduce the constitutive cytokine release by primary human AML cells [24,46,47]. These previous observations at the protein level are also consistent with present results describing an effect of NF-κB inhibition on cytokine-induced signaling. 

We used network analysis to further evaluate the possible biological relevance of our present observations, and we then identified four interacting clusters (see Figure 4 and Table S2). First, one of the networks included 10 genes, and previous studies have shown that all 10 encoded proteins are relevant for leukemogenesis and chemosensitivity in human AML, and may thus be possible therapeutic targets in human AML (see Table S2 for details and references). Second, another subset of genes encoded proteins that are important for mitochondrial functions, and thereby, probably also for the regulation of cellular metabolism. The targeting of mitochondria or cellular metabolism is now regarded as a possible therapeutic strategy in human AML [48,49,50,51]. Third, several downregulated genes encoded interacting proteins that are important for the spliceosome or RNA binding/transport. Genes encoding splicing factors could be mutated in human AML and these mutations seem to have an adverse prognostic impact [52,53]. RNA splicing/metabolism may, therefore, represent a possible therapeutic target in AML. Finally, a last network included genes that encode IFN-induced proteins; these proteins may also be important in AML [38]. Taken together, our present studies suggest that NF-κB inhibition inhibits several intracellular molecular mechanisms that now are regarded as possible therapeutic targets in future AML therapy. NF-κB inhibition may, therefore, represent inhibition of several therapeutic targets in human AML cells through the use of single therapeutic agents. However, protein expression is regulated and depends on several mechanisms and not only the regulation at the mRNA level, and for these reasons, our network analyses have to be interpreted with care. 

We aimed to classify the genes that showed altered expression after NF-κB inhibition, we then used well-recognized bioinformatical tools. Based on our 183 downregulated genes after NF-κB inhibition, we applied an enrichment analysis in search for the overrepresented GO-terms. We identified several terms, including terms representing functional classes of genes know to encode proteins that are important in leukemogenesis. Based on the analysis, three major biological systems seemed to be among the most influenced by NF-κB inhibition, including (i) cytokine and interleukin (IL) signaling, (ii) metabolic regulation, and (iii) immune system/cell communication. In the first class, i.e., cytokine and interleukin signaling, especially IL-4, IL-10, IL-13, and IFN seemed to be important. These observations are in accordance with previous studies describing a role for NF-κB in the regulation of constitutive cytokine release by primary human AML cells [16,54,55,56].

Several cytokines could be constitutively released by AML blasts [16] and are important for intercellular communication in the bone marrow microenvironment [29]. Metabolic targeting is now considered as a therapeutic strategy in human malignancies, including AML [57]. This is especially true for the 10–15% of AML patients with mutations in the metabolic genes isocitrate dehydrogenase (*IDH*) 1 or 2, resulting in increased levels of oncometabolites [58]. However, altered metabolic regulation seems to be important also in other AML subclasses [59]. Based on these considerations, it is interesting that metabolic regulators are targeted by NF-κB inhibition. This was most prominent when using the PANTHER GO-terms where the metabolic process was the term with the most differentially expressed genes. This is further supported by the fact that both mitochondrial function, adipocytes, and the central metabolic pathway Peroxisome proliferator-activated receptor α (PPARA) appear to be affected by NF-κB inhibition in AML based on the GO-mapping. Finally, the effect of NF-κB inhibition on genes involved in immunoregulation is also in accordance with previous AML studies [6], and with the initial discovery of NF-κB as an enhancer of B-cell functions [7]. These features are again related to the alterations in cytokine-associated genes, as chemokines and interleukins are important for the functions of immunocompetent cells. Finally, the importance of NF-κB in the transcriptional regulation of several genes involved in leukemogenesis is also reflected in our study of several single genes that were altered by NF-κB inhibition, e.g., *NFKB1*, *CXCL10*, and *IL1B* [5,6,29]. 

We identified nucleic acid binding, enzyme modulators, and transcription factors to be the three largest terms in our protein class analysis, and previous studies have shown that several of the downregulated genes are involved in leukemogenesis. First, several genes that are frequently mutated in AML were downregulated, including *RUNX1* and *CEBPA*, which are mutated in 10–15% and 5–10% of patients, respectively. Noteworthy, *RUNX1* mutations are associated with adverse prognosis [2] and are often detected in secondary AML [30]. In contrast, *CEBPA* mutations, at least in the case of double-strand mutations, are usually associated with chemosensitivity and favorable outcome [2,31]. (ii) Second, the expression of several genes encoding inflammatory cytokines (e.g., IL-1β, CXCL5, CXCL10, CCL2) were also significantly downregulated by NF-κB inhibition. These observations suggest that the effects of NF-κB inhibition on single cytokines should be regarded as a network effect [5,29], and our results confirmed that NF-κB is an important regulator of the cytokine network [16,29,60]. Finally, genes encoding central regulatory proteins involved in leukemogenesis were downregulated by NF-κB inhibition, including *BID*, encoding a proapoptotic member of the Bcl-2 protein family, and thereby, possibly being antagonistic to the Bcl-2 inhibitor venetoclax [61]. The Ras-related protein Ral-A (RALA) was also downregulated; this protein functions as a molecular switch to activate several fundamental biological processes of many different cancer cells [62]. Noteworthy is also the altered expression of *NFKB1* that encodes the Rel specific transcription inhibitor that is a part of the DNA binding subunit of the NF-κB complex [9,34]. Compensatory upregulation of this gene in response to NF-κB inhibition is probably consistent with this. Our network analysis also identified a network associated with NF-κB inhibition as well as altered expression of *MRRPL*, *SNRP*, and *IFIT* genes that possibly are involved in malignant cell transformation [35,36,37,38]. Of particular interest in the setting of AML is *SNRP* that is crucial for the spliceosome [37]; this effect may be relevant, especially for patients with mutations in spliceosome genes [4].

NF-κB inhibition could alter a 17-gene expression profile associated with LSCs. The elimination of LSCs by antileukemic therapy is probably of particular importance, since these cells are believed to be at the top of the hierarchy and be responsible for AML relapse [13]. They are also characterized by an LSC-specific gene expression signature [14]. Herein, we demonstrated that NF-κB inhibition could alter this transcriptomic signature, indicating a possible role for NF-κB inhibition in the targeting of the leukemic stem cells.

In the present study, we used the NF-κB inhibitor BMS-345541 that selectively interacts with the inhibitor of κB (IκB) kinase (IKK), and it is regarded as a highly selective inhibitor of NF-κB [6]. However, several agents could inhibit NF-κB function through various direct and indirect mechanisms. It should, therefore, be emphasized that the alterations in GEP described in this study might not be specific for NF-κB inhibition in AML cells, but should possibly be regarded as a model for the potential biological effects of NF-κB inhibition in cancer patients. In Table 2, we have summarized some in vitro studies of NF-κB inhibition in AML. Several of these studies describe antiproliferative, proapoptotic, antiangiogenic, or antimigratory effects of NF-κB inhibition. Furthermore, given the considerable heterogeneity between AML patients, additional effects may be detected for single patients or subsets of patients. Our study, including 16 patients, is too small to detect differences in NF-κB inhibition between patients, i.e., subsets defined by cytogenetic or molecular genetic alterations. Our study rather describes the effects that are common for all or most AML patients. 

## 5. Conclusions

NF-κB is an important transcription factor involved in AML leukemogenesis, and it is also regarded as a potential therapeutic target. However, the biological effects of NF-κB inhibition are only partly known, and to the best of our knowledge, this is the first study to describe the effects of NF-κB inhibition on the global gene expression profiles of primary AML cells derived from a group of consecutive and thereby unselected patients. We identified a significant and highly discriminative gene expression profile associated with NF-κB inhibition. We identified cytokine and interleukin signaling, alterations in metabolic systems, altered cellular communication, and transcriptional regulation among the altered cellular functions after NF-κB inhibition. Additional animal studies are needed before NF-κB inhibition can move into clinical studies, and BMS-345541 may not be the best inhibitor to be tried in the first clinical studies, but animal investigations have identified new agents that have NF-κB inhibitory effects and could be used as oral treatment [69,70]. 

## Figures and Tables

**Table 1:** Patient overview. Biological and demographical characteristics of the 16 patients included in the study.

Number	Gender	Age	Etiology	FAB	CD 34	Hb (g/dL)	Platelets (×10^9^/L)	Cytogenetics	*FLT3*	*NPM1*
1	F	87	*De novo*	M0	Pos	8.6	22	Del 5	wt	wt
2	M	65	*De novo*	M5	Neg	9.2	213	Normal	ITD	INS
3	M	39	*De novo*	M3	Pos	7.0	12	t(15;17)	wt	wt
4	M	78	MDS	nt	nt	8.3	30	Trisomy 11	nt	nt
5	M	63	*De novo*	M5	Neg	7.8	31	Normal	wt	ins
6	M	82	*De novo*	nt	Pos	11.8	50	Trisomy 8	wt	wt
7	F	77	MDS	M2	nt	10.7	35	Normal	ITD	INS
8	M	78	*De novo*	M1	Pos	8.3	206	Complex	nt	nt
9	M	84	*De novo*	M1	Pos	9.0	116	Complex	wt	wt
10	F	46	*De novo*	M1	Pos	10.8	1182	Inv(16)	wt	wt
11	M	46	*De novo*	M1	nt	15.4	113	Normal	wt	INS
12	F	82	Relapse	M2	Neg	9.2	16	Normal	ITD	INS
13	M	42	*De novo*	M5	Neg	10.6	179	Normal	ITD	INS
14	F	59	*De novo*	M4	Neg	nt	nt	Normal	ITD	INS
15	M	78	*De novo*	M1	Pos	9.5	35	Normal	wt	wt
16	M	75	CMML	nt	nt	10.8	44	Del 12p, Del 7	nt	nt

Abbreviations: CMML, chronic myelomonocytic leukemia; Del, deletion; F, female; FAB, French-American-British; Hb, hemoglobin; INS, insertion; ITD internal tandem duplication; Inv, inversion; M, male: MDS, myelodysplastic syndrome; Neg, negative; nt, not tested Pos, positive; wt, wild type.

**Table 2:** Summary of important in vitro studies of NF-κB inhibition in AML.

Drug	Patients/AML Cell Lines	Major Findings	References
AS602868	61 patients	Induced cytotoxicity and alteration in GEP, especially effects on immune-related genes.	[63]
Xanthumol	U937 and HL-60 cell lines	Suppression of antiapoptotic gene expression, increased apoptosis in leukemia cells.	[64]
Reseveratrol	OCIM2 and OCI-AML3 cell lines, five patient samples	Proapoptotic and antiproliferative effects in suspension cultures and colony-formation assays, decreased constitutive cytokine release	[65]
Curcumin	SHI1 cell lines	Inhibition of proliferation and cell migration, altered transcriptional regulation, decreased cytokine secretion.	[66]
Celastrol	HL-60 cell line	Antiproliferative and proapoptotic effects	[67]
BAY 11-7082	OCI-AML3 cell line	Antiproliferative and antiangiogenic effects	[68]

## Supplementary Materials

The following are available online at https://www.mdpi.com/2073-4409/9/7/1677/s1. Table S1. Gene differently expressed by NF-κB inhibition in primary AML cells. Table S2. Protein interaction networks modulated by NF-κB inhibition in human AML; a summary of the four interaction networks showing decreased mRNA expression in primary human AML in response to NF-κB inhibition. Table S3. Protein interaction networks modulated by NF-κB inhibition in human AML; a summary of interacting genes with showing increased mRNA expression in primary human AML in response to NF-κB inhibition. Figure S1. Network analysis of genes with upregulated expression after NF-κB inhibition.

## References

[1] Döhner H., Weisdorf D.J., Bloomfield C.D. (2015). Acute Myeloid Leukemia. N. Engl. J. Med..

[2] Döhner H., Estey E., Grimwade D., Amadori S., Appelbaum F.R., Büchner T., Dombret H., Ebert B.L., Fenaux P., Larson R.A. (2017). Diagnosis and management of AML in adults: 2017 ELN recommendations from an international expert panel. Blood.

[3] Ossenkoppele G., Löwenberg B. (2015). How I treat the older patient with acute myeloid leukemia. Blood.

[4] Papaemmanuil E., Gerstung M., Bullinger L., Gaidzik V.I., Paschka P., Roberts N.D., Potter N.E., Heuser M., Thol F., Bolli N. (2016). Genomic Classification and Prognosis in Acute Myeloid Leukemia. N. Engl. J. Med..

[5] Reikvam H., Aasebø E., Brenner A.K., Bartaula-Brevik S., Grønningsæter I.S., Forthun R.B., Hovland R., Bruserud Ø. (2019). High Constitutive Cytokine Release by Primary Human Acute Myeloid Leukemia Cells Is Associated with a Specific Intercellular Communication Phenotype. J. Clin. Med..

[6] Reikvam H., Olsnes A.M., Gjertsen B.T., Ersvar E., Bruserud Ø. (2009). Nuclear factor-kappaB signaling: A contributor in leukemogenesis and a target for pharmacological intervention in human acute myelogenous leukemia. Crit. Rev. Oncog..

[7] Sen R., Baltimore D. (1986). Multiple nuclear factors interact with the immunoglobulin enhancer sequences. Cell.

[8] Xia L., Tan S., Zhou Y., Lin J., Wang H., Oyang L., Tian Y., Liu L., Su M., Wang H. (2018). Role of the NFkappaB-signaling pathway in cancer. Onco Targets.

[9] Paul A., Edwards J., Pepper C., Mackay S. (2018). Inhibitory-kappaB Kinase (IKK) alpha and Nuclear Factor-kappaB (NFkappaB)-Inducing Kinase (NIK) as Anti-Cancer Drug Targets. Cells.

[10] Bosman M.C., Schuringa J.J., Vellenga E. (2016). Constitutive NF-kappaB activation in AML: Causes and treatment strategies. Crit. Rev. Oncol. Hematol..

[11] Guzman M.L., Neering S.J., Upchurch D., Grimes B., Howard D.S., Rizzieri D.A., Luger S.M., Jordan C.T. (2001). Nuclear factor-κB is constitutively activated in primitive human acute myelogenous leukemia cells. Blood.

[12] Eppert K., Takenaka K., Lechman E.R., Waldron L.D., Nilsson B., Van Galen P., Metzeler K.H., Poeppl A., Ling V., Beyene J. (2011). Stem cell gene expression programs influence clinical outcome in human leukemia. Nat. Med..

[13] Bruserud Ø., Aasebø E., Hernandez-Valladares M., Tsykunova G., Reikvam H. (2017). Therapeutic targeting of leukemic stem cells in acute myeloid leukemia—The biological background for possible strategies. Expert Opin. Drug Discov..

[14] Ng S.W.K., Mitchell A., Kennedy J.A., Chen W.C., McLeod J., Ibrahimova N., Arruda A., Popescu A., Gupta V., Schimmer A.D. (2016). A 17-gene stemness score for rapid determination of risk in acute leukaemia. Nature.

[15] Reikvam H., Brenner A.K., Hagen K.M., Liseth K., Skrede S., Hatfield K., Bruserud Ø. (2015). The cytokine-mediated crosstalk between primary human acute myeloid cells and mesenchymal stem cells alters the local cytokine network and the global gene expression profile of the mesenchymal cells. Stem Cell Res..

[16] Bruserud Ø., Ryningen A., Olsnes A.M., Stordrange L., Øyan A.M., Kalland K.H., Gjertsen B.T. (2007). Subclassification of patients with acute myelogenous leukemia based on chemokine responsiveness and constitutive chemokine release by their leukemic cells. Haematologica.

[17] Gilmore T.D., Herscovitch M. (2006). Inhibitors of NF-kappaB signaling: 785 and counting. Oncogene.

[18] Burke J.R., Pattoli M.A., Gregor K.R., Brassil P.J., MacMaster J.F., McIntyre K.W., Yang X., Iotzova V.S., Clarke W., Strnad J. (2003). BMS-345541 is a highly selective inhibitor of I kappa B kinase that binds at an allosteric site of the enzyme and blocks NF-kappa B-dependent transcription in mice. J. Biol. Chem..

[19] Yang J., Amiri K.I., Burke J.R., Schmid J.A., Richmond A. (2006). BMS-345541 targets inhibitor of kappaB kinase and induces apoptosis in melanoma: Involvement of nuclear factor kappaB and mitochondria pathways. Clin. Cancer Res..

[20] Battula V.L., Nguyen K., Sun J., Pitner M.K., Yuan B., Bartholomeusz C., Hail N., Andreeff M. (2017). IKK inhibition by BMS-345541 suppresses breast tumorigenesis and metastases by targeting GD2+ cancer stem cells. Oncotarget.

[21] Döhner H., Estey E.H., Amadori S., Appelbaum F.R., Buchner T., Burnett A.K., Dombret H., Fenaux P., Grimwade D., Larson R.A. (2010). Diagnosis and management of acute myeloid leukemia in adults: Recommendations from an international expert panel, on behalf of the European LeukemiaNet. Blood.

[22] Bruserud Ø., Hovland R., Wergeland L., Huang T.-S., Gjertsen B.T. (2003). Flt3-mediated signaling in human acute myelogenous leukemia (AML) blasts: A functional characterization of Flt3-ligand effects in AML cell populations with and without genetic Flt3 abnormalities. Haematologica.

[23] Reikvam H., Nepstad I., Bruserud Ø., Hatfield K.J. (2013). Pharmacological targeting of the PI3K/mTOR pathway alters the release of angioregulatory mediators both from primary human acute myeloid leukemia cells and their neighboring stromal cells. Oncotarget.

[24] Reikvam H., Hatfield K., Lassalle P., Kittang A.O., Ersvær E., Bruserud Ø. (2010). Targeting the angiopoietin (Ang)/Tie-2 pathway in the crosstalk between acute myeloid leukaemia and endothelial cells: Studies of Tie-2 blocking antibodies, exogenous Ang-2 and inhibition of constitutive agonistic Ang-1 release. Expert Opin. Investig. Drugs.

[25] Reikvam H., Tamburini J., Skrede S., Holdhus R., Poulain L., Ersvaer E., Hatfield K., Bruserud Ø., Ersvær E. (2013). Antileukaemic effect of PI3K-mTOR inhibitors in acute myeloid leukaemia-gene expression profiles reveal CDC25B expression as determinate of pharmacological effect. Br. J. Haematol..

[26] Stavrum A.-K., Petersen K., Jonassen I., Dysvik B. (2008). Analysis of Gene-Expression Data Using J-Express. Curr. Protoc. Bioinform..

[27] Szklarczyk D., Morris J.H., Cook H.V., Kuhn M., Wyder S., Simonovic M., Santos A., Doncheva N.T., Roth A., Bork P. (2016). The STRING database in 2017: Quality-controlled protein-protein association networks, made broadly accessible. Nucleic Acids Res..

[28] Mi H., Muruganujan A., Ebert D., Huang X., Thomas P. (2018). PANTHER version 14: More genomes, a new PANTHER GO-slim and improvements in enrichment analysis tools. Nucleic Acids Res..

[29] Reikvam H., Hatfield K.J., Fredly H., Nepstad I., Mosevoll K.A., Bruserud Ø. (2012). The angioregulatory cytokine network in human acute myeloid leukemia – from leukemogenesis via remission induction to stem cell transplantation. Eur. Cytokine Netw..

[30] Gaidzik V.I., Bullinger L., Schlenk R.F., Zimmermann A.S., Röck J., Paschka P., Corbacioglu A., Krauter J., Schlegelberger B., Ganser A. (2011). RUNX1 Mutations in Acute Myeloid Leukemia: Results From a Comprehensive Genetic and Clinical Analysis From the AML Study Group. J. Clin. Oncol..

[31] Taskesen E., Bullinger L., Corbacioglu A., Sanders M.A., Erpelinck C.A.J., Wouters B.J., Luytgaarde S.C.V.D.P.-V.D., Damm F., Krauter J., Ganser A. (2011). Prognostic impact, concurrent genetic mutations, and gene expression features of AML with CEBPA mutations in a cohort of 1182 cytogenetically normal AML patients: Further evidence for CEBPA double mutant AML as a distinctive disease entity. Blood.

[32] Gonçalves A.C., Alves R., Baldeiras I., Cortesão E., Carda J.P., Branco C.C., Oliveiros B., Loureiro L., Pereira A., Costa J.M.N. (2016). Genetic variants involved in oxidative stress, base excision repair, DNA methylation, and folate metabolism pathways influence myeloid neoplasias susceptibility and prognosis. Mol. Carcinog..

[33] Gu C., Feng M., Yin Z., Luo X., Yang J., Li Y., Li T., Wang R., Fei J. (2016). RalA, a GTPase targeted by miR-181a, promotes transformation and progression by activating the Ras-related signaling pathway in chronic myelogenous leukemia. Oncotarget.

[34] Concetti J., Wilson C.L. (2018). NFKB1 and Cancer: Friend or Foe?. Cells.

[35] Sotgia F., Fiorillo M., Lisanti M.P. (2017). Mitochondrial markers predict recurrence, metastasis and tamoxifen-resistance in breast cancer patients: Early detection of treatment failure with companion diagnostics. Oncotarget.

[36] Lee Y.-K., Lim J.J., Jeoun U.-W., Min S., Lee E.-B., Kwon S.M., Lee C., Yoon G. (2017). Lactate-mediated mitoribosomal defects impair mitochondrial oxidative phosphorylation and promote hepatoma cell invasiveness. J. Boil. Chem..

[37] Chari A., Golas M.M., Klingenhäger M., Neuenkirchen N., Sander B., Englbrecht C., Sickmann A., Stark H., Fischer U. (2008). An Assembly Chaperone Collaborates with the SMN Complex to Generate Spliceosomal SnRNPs. Cell.

[38] Pidugu V.K., Pidugu H.B., Wu M.-M., Liu C.-J., Lee T.-C. (2019). Emerging Functions of Human IFIT Proteins in Cancer. Front. Mol. Biosci..

[39] Verhagen H.J., Van Gils N., Martiañez T., Van Rhenen A., Rutten A., Denkers F., De Leeuw D.C., Smit M.A., Tsui M.-L., Klootwijk L.L.D.V. (2018). IGFBP7 Induces Differentiation and Loss of Survival of Human Acute Myeloid Leukemia Stem Cells without Affecting Normal Hematopoiesis. Cell Rep..

[40] Wu L., Amarachintha S., Xu J., Oley F., Du W. (2018). Mesenchymal COX2-PG secretome engages NR4A-WNT signalling axis in haematopoietic progenitors to suppress anti-leukaemia immunity. Br. J. Haematol..

[41] Valk P.J.M., Verhaak R.G.W., Beijen M.A., Erpelinck C.A., Doorn-Khosrovani S.B.V.W.V., Boer J.M., Beverloo H.B., Moorhouse M.J., Van Der Spek P., Löwenberg B. (2004). Prognostically Useful Gene-Expression Profiles in Acute Myeloid Leukemia. N. Engl. J. Med..

[42] Ley T.J., Miller C., Ding L., Raphael B.J., Mungall A.J., Robertson A.G., Hoadley K.A., Triche T.J., Laird P.W., Cancer Genome Atlas Research Network (2013). Genomic and epigenomic landscapes of adult de novo acute myeloid leukemia. N. Engl. J. Med..

[43] Hinz M., Lemke P., Anagnostopoulos I., Hacker C., Krappmann D., Mathas S., Dörken B., Zenke M., Stein H., Scheidereit C. (2002). Nuclear factor kappaB-dependent gene expression profiling of Hodgkin’s disease tumor cells, pathogenetic significance, and link to constitutive signal transducer and activator of transcription 5a activity. J. Exp. Med..

[44] Bullinger L., Döhner K., Bair E., Fröhling S., Schlenk R.F., Tibshirani R., Döhner H., Pollack J.R. (2004). Use of Gene-Expression Profiling to Identify Prognostic Subclasses in Adult Acute Myeloid Leukemia. N. Engl. J. Med..

[45] Carvalho G., Fabre C., Braun T., Grosjean J., Ades L., Agou F., Tasdemir E., Boehrer S., Israel A., Veron M. (2006). Inhibition of NEMO, the regulatory subunit of the IKK complex, induces apoptosis in high-risk myelodysplastic syndrome and acute myeloid leukemia. Oncogene.

[46] Reikvam H., Hatfield K.J., Øyan A.M., Kalland K.H., Kittang A.O., Bruserud Ø. (2010). Primary human acute myelogenous leukemia cells release matrix metalloproteases and their inhibitors: Release profile and pharmacological modulation. Eur. J. Haematol..

[47] Olsnes A.M., Ersvaer E., Ryningen A., Paulsen K., Hampson P., Lord J., Gjertsen B.T., Kristoffersen E.K., Bruserud Ø. (2009). The protein kinase C agonist PEP005 increases NF-κB expression, induces differentiation and increases constitutive chemokine release by primary acute myeloid leukaemia cells. Br. J. Haematol..

[48] Basak N.P., Banerjee S. (2015). Mitochondrial dependency in progression of acute myeloid leukemia. Mitochondrion.

[49] Aasebø E., Berven F.S., Hovland R., Døskeland S., Bruserud Ø., Selheim F., Hernandez-Valladares M. (2020). The Progression of Acute Myeloid Leukemia from First Diagnosis to Chemoresistant Relapse: A Comparison of Proteomic and Phosphoproteomic Profiles. Cancers.

[50] Panina S.B., Pei J., Baran N., Konopleva M., Kirienko N.V. (2020). Utilizing Synergistic Potential of Mitochondria-Targeting Drugs for Leukemia Therapy. Front. Oncol..

[51] Grønningsæter I.S., Reikvam H., Aasebø E., Bartaula-Brevik S., Tvedt T.H., Bruserud Ø., Hatfield K.J. (2020). Targeting Cellular Metabolism in Acute Myeloid Leukemia and the Role of Patient Heterogeneity. Cells.

[52] De Necochea-Campion R., Shouse G.P., Zhou Q., Mirshahidi S., Chen C.-S. (2016). Aberrant splicing and drug resistance in AML. J. Hematol. Oncol..

[53] Zhou J., Chng W.-J. (2017). Aberrant RNA splicing and mutations in spliceosome complex in acute myeloid leukemia. Stem Cell Investig..

[54] Zhou J., Ching Y.Q., Chng W.-J. (2015). Aberrant nuclear factor-kappa B activity in acute myeloid Leukemia: From molecular pathogenesis to therapeutic target. Oncotarget.

[55] Li J., Volk A., Zhang J., Cannova J., Dai S., Hao C., Hu C., Sun J., Xu Y., Wei W. (2016). Sensitizing leukemia stem cells to NF-κB inhibitor treatment in vivo by inactivation of both TNF and IL-1 signaling. Oncotarget.

[56] Volk A., Li J., Xin J., You D., Zhang J., Liu X., Xiao Y., Breslin P., Li Z., Wei W. (2014). Co-inhibition of NF-kappaB and JNK is synergistic in TNF-expressing human AML. J. Exp. Med.

[57] Grønningsæter I.S., Fredly H.K., Gjertsen B.T., Hatfield K.J., Bruserud Ø. (2019). Systemic Metabolomic Profiling of Acute Myeloid Leukemia Patients before and During Disease-Stabilizing Treatment Based on All-Trans Retinoic Acid, Valproic Acid, and Low-Dose Chemotherapy. Cells.

[58] Marcucci G., Maharry K., Wu Y.-Z., Radmacher M.D., Mrózek K., Margeson D., Holland K.B., Whitman S.P., Becker H., Schwind S. (2010). IDH1andIDH2Gene Mutations Identify Novel Molecular Subsets Within De Novo Cytogenetically Normal Acute Myeloid Leukemia: A Cancer and Leukemia Group B Study. J. Clin. Oncol..

[59] Nepstad I., Hatfield K.J., Grønningsæter I.S., Reikvam H. (2020). The PI3K-Akt-mTOR Signaling Pathway in Human Acute Myeloid Leukemia (AML) Cells. Int. J. Mol. Sci..

[60] Berdyshev A.G., Kosiakova H.V., Onopchenko O.V., Panchuk R.R., Stoika R.S., Hula N.M. (2015). N-Stearoylethanolamine suppresses the pro-inflammatory cytokines production by inhibition of NF-kappaB translocation. Prostaglandins Other Lipid Mediat..

[61] Dinardo C.D., Pratz K., Pullarkat V., Jonas B., Arellano M., Becker P.S., Frankfurt O., Konopleva M., Wei A.H., Kantarjian H.M. (2019). Venetoclax combined with decitabine or azacitidine in treatment-naive, elderly patients with acute myeloid leukemia. Blood.

[62] Moghadam A.R., Patrad E., Tafsiri E., Peng W., Fangman B., Pluard T.J., Accurso A., Salacz M., Shah K., Ricke B. (2017). Ral signaling pathway in health and cancer. Cancer Med..

[63] Jordheim L.P., Plesa A., Dreano M., Cros-Perrial E., Keime C., Herveau S., Demangel D., Vendrell J.A., Dumontet C. (2010). Sensitivity and gene expression profile of fresh human acute myeloid leukemia cells exposed ex vivo to AS602868. Cancer Chemother. Pharmacol..

[64] Harikumar K.B., Kunnumakkara A.B., Ahn K.S., Anand P., Krishnan S., Guha S., Aggarwal B.B. (2009). Modification of the cysteine residues in IkappaBalpha kinase and NF-kappaB (p65) by xanthohumol leads to suppression of NF-kappaB-regulated gene products and potentiation of apoptosis in leukemia cells. Blood.

[65] Estrov Z., Shishodia S., Faderl S., Harris D., Van Q., Kantarjian H.M., Talpaz M., Aggarwal B.B. (2003). Resveratrol blocks interleukin-1beta-induced activation of the nuclear transcription factor NF-kappaB, inhibits proliferation, causes S-phase arrest, and induces apoptosis of acute myeloid leukemia cells. Blood.

[66] Zhu G.-H., Dai H.-P., Shen Q., Ji O., Zhang Q., Zhai Y.-L. (2015). Curcumin induces apoptosis and suppresses invasion through MAPK and MMP signaling in human monocytic leukemia SHI-1 cells. Pharm. Boil..

[67] Jaliani H.Z., Pazhang Y., Imani M., Dariushnejad H. (2016). Synergism between NF-kappa B inhibitor, celastrol, and XIAP inhibitor, embelin, in an acute myeloid leukemia cell line, HL-60. J. Cancer Res. Ther..

[68] Omsland M., Bruserud O., Gjertsen B.T., Andresen V. (2017). Tunneling nanotube (TNT) formation is downregulated by cytarabine and NF-kappaB inhibition in acute myeloid leukemia (AML). Oncotarget.

[69] Guzman M.L., Rossi R.M., Neelakantan S., Li X., Corbett C.A., Hassane D., Becker M., Bennett J.M., Sullivan E., Lachowicz J.L. (2007). An orally bioavailable parthenolide analog selectively eradicates acute myelogenous leukemia stem and progenitor cells. Blood.

[70] Guzman M.L., Rossi R.M., Karnischky L., Li X., Peterson D.R., Howard D.S., Jordan C.T. (2005). The sesquiterpene lactone parthenolide induces apoptosis of human acute myelogenous leukemia stem and progenitor cells. Blood.

