# Supplementary material for: Inhibition of NF-κB Signaling Alters Acute Myelogenous Leukemia Cell Transcriptomics
Source: Cells. 2020 Jul 12;9(7):1677. doi: 10.3390/cells9071677 (PMC7408594; doi:10.3390/cells9071677)
Supplement: Supplementary file 1 [file cells-09-01677-s001.pdf]

SUPPLEMENTARY INFORMATION

Inhibition of NF-κB signaling alters acute myelogenous leukemia cell transcriptomics

Håkon Reikvam [1,2]

[1] Institute of Clinical Science, University of Bergen, Bergen, Norway; Hakon.Reikvam@uib.no
[2] Department of Medicine, Haukeland University Hospital, Bergen, Norway
* Correspoence: Professor Håkon Reikvam; Hakon.Reikvam@uib.no

Table S1. Down- and upregulated genes in response to NF-κB inhibition in AML.

Downregulatated genes		
**Gen ID**	**Probe ID**	**Symbol**
13286	ILMN_1718537	HPS6
30823	ILMN_1742789	LPXN
17085	ILMN_1775501	IL1B
7086	ILMN_1791759	CXCL10
17286	ILMN_1793474	INSIG1
11473	ILMN_1675191	GAPT
33801	ILMN_1737738	NDUFA12
43560	ILMN_1770338	TM4SF1
5549	ILMN_1784602	CDKN1A
17204	ILMN_1733696	IMP3
43861	ILMN_1752213	TMEM60
5099	ILMN_1720048	CCL2
42188	ILMN_2041293	SQLE
38317	ILMN_1755364	RALA
16880	ILMN_1739428	IFIT2
45555	ILMN_1705310	VEZF1
38915	ILMN_1739792	RHOG
17378	ILMN_2394561	IRF2BP2
2478	ILMN_1776181	BIRC3
11474	ILMN_3242271	GAPT
38130	ILMN_1793433	RAB10
43384	ILMN_1686116	THBS1
33269	ILMN_2073289	MTSS1
33970	ILMN_1714965	NFKB1
18668	ILMN_1703335	LACTB
4293	ILMN_1739798	C7orf30
1024	ILMN_1793894	ANAPC13
8529	ILMN_1798706	EBI2
39624	ILMN_1801504	RUNX1
21982	ILMN_3244323	LOC148413
29313	ILMN_1692072	LOC728006
9313	ILMN_1708936	EXOSC3
5325	ILMN_1803429	CD44
3092	ILMN_1661945	C14orf156
8879	ILMN_1765446	EMP3
36892	ILMN_2147306	PNRC2
5623	ILMN_1715715	CEBPA
4014	ILMN_1690454	C3orf54
13088	ILMN_1657395	HMGCR
41744	ILMN_1683562	SNRPG

2710	ILMN_1775743	BTG1
31588	ILMN_1672660	MBP
18646	ILMN_1746517	KYNU
46178	ILMN_1694466	ZBED1
32946	ILMN_1689774	MRFAP1L1
7158	ILMN_1745256	CXXC5
32978	ILMN_1797933	MRPL17
42191	ILMN_1662618	SQSTM1
42265	ILMN_1796407	SS18L2
44394	ILMN_2323385	TRIM4
1436	ILMN_1699703	ARCN1
33379	ILMN_2219767	MYCN
40373	ILMN_1734895	SFT2D1
34629	ILMN_1738681	NUP62
32979	ILMN_1804479	MRPL18
39499	ILMN_1783709	RRAGA
39738	ILMN_1814305	SAMD9
16877	ILMN_1707695	IFIT1
47251	ILMN_1812856	ZSWIM1
9402	ILMN_1758658	FADD
33005	ILMN_2341952	MRPL35
19965	ILMN_3256004	LOC100130003
39535	ILMN_1791097	RSBN1
7223	ILMN_1693338	CYP1B1
5650	ILMN_1664028	CENPB
16685	ILMN_2413084	HSPA8
37192	ILMN_1747598	PPP1R11
9268	ILMN_1763452	EVI2B
36168	ILMN_1678546	PEX11B
1393	ILMN_2149766	APPBP2
32988	ILMN_1663220	MRPL22
39444	ILMN_1712678	RPS27L
16882	ILMN_1701789	IFIT3
7159	ILMN_3307729	CXXC5
13089	ILMN_1797728	HMGCS1
5017	ILMN_2064898	CCDC56
37738	ILMN_1759952	PSMA5
16920	ILMN_1764964	IFNGR2
38807	ILMN_1654398	RGL1
6950	ILMN_1692962	CTDSP2
37571	ILMN_1748908	PROSC
35905	ILMN_1671621	PCMT1
43792	ILMN_2073012	TMEM203
18647	ILMN_1737514	KYNU
31826	ILMN_1691570	METTL5

18426	ILMN_1664756	KPNA4
8946	ILMN_2166865	ENY2
12710	ILMN_1803945	HCP5
37807	ILMN_2088410	PSMG2
1567	ILMN_1721626	ARID5B
16686	ILMN_1686367	HSPA8
31883	ILMN_2342240	MGAT2
43851	ILMN_1674985	TMEM51
4629	ILMN_1687858	CALM2
43791	ILMN_2073010	TMEM203
8528	ILMN_2168217	EBI2
36751	ILMN_2138765	PLIN2
36148	ILMN_1770811	PELO
38222	ILMN_1716524	RAB7A
5845	ILMN_1679428	CHIC2
32984	ILMN_1693352	MRPL20
31819	ILMN_2343624	METTL13
40246	ILMN_2150851	SERPINB2
6001	ILMN_1712389	CKLF
23134	ILMN_3200330	LOC399988
6453	ILMN_2300396	COMMD5
31181	ILMN_1764709	MAFB
34690	ILMN_1674063	OAS2
16876	ILMN_1781373	IFIH1
44020	ILMN_2112256	TNFRSF4
35526	ILMN_1722276	PAFAH1B1
11088	ILMN_1788213	FRAT2
44293	ILMN_1814650	TRAPPC4
33810	ILMN_1759729	NDUFA8
32975	ILMN_2072603	MRPL14
31710	ILMN_1777526	MED20
34046	ILMN_1815086	NINJ1
42420	ILMN_1740819	STARD7
31522	ILMN_1811367	MAT2B
5337	ILMN_2147517	CD58
43370	ILMN_1780699	THAP11
6519	ILMN_2187718	COX17
4243	ILMN_1795286	C6orf47
11826	ILMN_1737308	GLRX
38227	ILMN_1750409	RAB9A
42479	ILMN_1655163	STK24
4352	ILMN_1760400	C8orf41
36056	ILMN_1739274	PDHB
2711	ILMN_1770085	BTG2
43640	ILMN_1712035	TMEM115

42189	ILMN_1772241	SQLE
33033	ILMN_1664833	MRPL50
32694	ILMN_1718718	MKKS
34785	ILMN_1723035	OLR1
36833	ILMN_1728660	PMPCB
38154	ILMN_1786976	RAB22A
5324	ILMN_2348788	CD44
44982	ILMN_2191428	UBB
6070	ILMN_1710326	CLDND1
41834	ILMN_2336781	SOD2
5989	ILMN_1663092	CITED2
3225	ILMN_2194828	C16orf53
33008	ILMN_1800197	MRPL36
11570	ILMN_1666545	GCNT1
6000	ILMN_2414027	CKLF
6069	ILMN_2352563	CLDND1
40603	ILMN_1758146	SIRPA
33959	ILMN_1790909	NFE2L2
12659	ILMN_1693826	HAVCR2
13098	ILMN_1700518	HMGN4
8091	ILMN_1793770	DNAJB6
18844	ILMN_3249748	LDHA
8704	ILMN_1669394	EI24
10194	ILMN_2144088	FDFT1
45637	ILMN_1761721	VPS35
5624	ILMN_1693014	CEBPB
43651	ILMN_2217809	TMEM126A
794	ILMN_1713124	AKR1C3
33606	ILMN_2354140	NAT5
43333	ILMN_2384241	TGFBR2
43991	ILMN_2414325	TNFAIP8
31521	ILMN_1737298	MAT2A
44225	ILMN_1727479	TPRG1L
9314	ILMN_1734194	EXOSC3
3173	ILMN_1793729	C15orf39
45630	ILMN_2344850	VPS26A
16199	ILMN_1906187	
39956	ILMN_1735360	SDAD1
2185	ILMN_1685824	B4GALT5
2467	ILMN_1763386	BID
4525	ILMN_1765858	CAB39
20488	ILMN_3278070	LOC100131336
33340	ILMN_1662358	MX1
1423	ILMN_1715068	AQP9
44983	ILMN_1762436	UBB

31726	ILMN_1671603	MED30
38926	ILMN_3242586	RHOU
12946	ILMN_1651496	HIST1H2BD
32980	ILMN_2230672	MRPL18
11729	ILMN_1776678	GIMAP7
39493	ILMN_1730077	RPUSD2
44016	ILMN_1764788	TNFRSF1B
33604	ILMN_1689097	NAT5

**Upregulatated genes**		
**Gen ID**	**Probe ID**	**Symbol**
1846	ILMN_2374865	ATF3
23616	ILMN_3283449	LOC440991
41536	ILMN_3238955	SNORD10
44295	ILMN_1775703	TRAPPC6A
30419	ILMN_3235013	LOC730288
39298	ILMN_2130180	RPL13L
21735	ILMN_3241834	LOC100134504
5548	ILMN_1787212	CDKN1A
33185	ILMN_1686664	MT2A
41479	ILMN_3241798	SNORA41
41435	ILMN_2189842	SNORA10
24569	ILMN_1655710	LOC642989
22806	ILMN_3280565	LOC389342
30741	ILMN_1691053	LOC91561
39202	ILMN_3241878	RNU6ATAC
20893	ILMN_3249578	LOC100132394
39384	ILMN_1689725	RPLP1
20677	ILMN_3199974	LOC100131787
39292	ILMN_3226244	RPL12P6
31849	ILMN_1756071	MFGE8
23742	ILMN_3280020	LOC441506
24490	ILMN_3289352	LOC642828
29201	ILMN_3211887	LOC727821
14652	ILMN_1816342	
15220	ILMN_1908824	
34390	ILMN_1782305	NR4A2
41430	ILMN_3238785	SNHG9
22555	ILMN_1786359	LOC346950
17608	ILMN_1782247	KAT2A
36884	ILMN_1662587	PNPLA7

997	ILMN_1665331	AMT
21687	ILMN_3246805	LOC100134364
30864	ILMN_1718633	LRP5L
30077	ILMN_3229570	LOC729500
35494	ILMN_3303612	PABPC1L
12986	ILMN_1732071	HIST2H2BE
16974	ILMN_2062468	IGFBP7
39207	ILMN_3246433	RNY5
41519	ILMN_3240418	SNORA72
212	ILMN_3187680	ACCS
19111	ILMN_1733559	LOC100008589
41691	ILMN_3244157	SNORD83B
44566	ILMN_1748124	TSC22D3
41459	ILMN_3235096	SNORA28
40095	ILMN_1724422	SELL
41467	ILMN_2096747	SNORA33
41626	ILMN_1667609	SNORD35A
15975	ILMN_1902251	
22409	ILMN_1795835	LOC338758
19110	ILMN_3243593	LOC100008588
44204	ILMN_1789196	TPM2
44206	ILMN_1757604	TPM2
826	ILMN_1782939	ALB
39188	ILMN_1768139	RNU12
21597	ILMN_3248649	LOC100134144
28890	ILMN_1695435	LOC653610
44565	ILMN_2376403	TSC22D3
39000	ILMN_3234762	RN5S9
18210	ILMN_1732988	KIAA1666
44473	ILMN_1776125	TRK1
39299	ILMN_1726460	RPL14
31271	ILMN_1721349	MAGT1
39852	ILMN_1805064	SCARNA9
41627	ILMN_1694367	SNORD35B
39201	ILMN_3310351	RNU6-15
41613	ILMN_1774973	SNORD21
39200	ILMN_3308335	RNU6-1
39195	ILMN_3309453	RNU4-1
39002	ILMN_2074860	RN7SK
39198	ILMN_3237617	RNU5A
18276	ILMN_1667594	KLF10
41596	ILMN_1892403	SNORD13
41509	ILMN_3247018	SNORA67
41512	ILMN_1730773	SNORA70
41692	ILMN_3245672	SNORD84

19112	ILMN_3251587	LOC100008589
39398	ILMN_1704056	RPPH1
41634	ILMN_3239574	SNORD3A
39196	ILMN_3308138	RNU4-2
41635	ILMN_3241034	SNORD3C
39191	ILMN_3245678	RNU1A3
41482	ILMN_3238078	SNORA45
41597	ILMN_1799381	SNORD14A
39192	ILMN_3240220	RNU1F1
15632	ILMN_1901419	
20966	ILMN_3243644	LOC100132564
39190	ILMN_3236653	RNU1-5
41636	ILMN_3242315	SNORD3D
39187	ILMN_3245103	RNU11
39001	ILMN_1739423	RN7SK
39189	ILMN_3246273	RNU1-3
39193	ILMN_3244646	RNU1G2
39197	ILMN_3240594	RNU4ATAC
10985	ILMN_1751607	FOSB

Table S2. Protein interaction networks modulated by NF-κB inhibition in human AML; a summary of the four interaction networks showing decreased mRNA expression in primary human AML in response to NF-κB inhibition (see Figure 4). The table is based on the Gene database and selected references included in the PubMed database.

PROTEIN	COMMENT	KEY WORDS
NETWORK 1 (leukemogenesis-chemosensitivity). *The encoded proteins in this cluster have all been linked to leukemogenesis and/or chemosensitivity in human AML. Several of them are important for transcriptional regulation, e.g. directly acting as transcription factors, epigenetic regulators or regulators of NF-κB.*		
CEBPA	*CCAAT enhancer binding protein alpha.* This transcription factor functions in homodimers and also heterodimers with CCAAT/enhancer-binding proteins beta and gamma. Activity of this protein can modulate the expression of genes involved in cell cycle regulation. Mutations of this gene are seen in human AML.	Transcription<br>Cell cycle regulation<br>AML mutation
CEBPB	*CCAAT enhancer binding protein beta.* This transcription factor that functions as a homodimer but can also form heterodimers with CCAAT/enhancer-binding proteins alpha, delta, and gamma. It is a downstream target of NFκB via the PU.1 tumor suppressor (PMID 19966862), and it is also important for chemosentivity and regulation of apoptosis in human AML (PMID 20981511)	Transcription<br>Apoptosis
IL1B	*Interleukin 1β.* This cytokine is a growth factor for and can be constitutively released by primary human AML cells (PMID 8632679).	AML growth factor
RRAGA	*Ras related GTP binding A.* Experimental studies suggest that targeting of this molecule can sensitize primary human AML cells to antileukemic treatment (PMID 31780813).	Chemosensitization
NFKB1	*Nuclear factor kappa B subunit 1.* This 105 kD protein can undergo cotranslational processing by the 26S proteasome to produce a 50 kD protein. It is a Rel protein-specific transcription inhibitor and the 50 kD protein is a DNA binding subunit of the NF-κB protein complex.	NF-κB
SQSTM1	*Sequestosome 1.* This multifunctional protein binds ubiquitin and regulates activation of NF-κB) signaling pathway. It mediates activation of NF-kB in response to upstream signals.	NF-κB
UBB	*Ubiquitin B.* Ubiquitin has a major role in targeting cellular proteins for degradation by the 26S proteasome. It is also involved in the maintenance of chromatin structure, the regulation of gene expression, and the stress response. Proteasome inhibitors are now tried in the treatment of AML (PMID 31379016, 19811823), and the proteasomes are in addition important for activation of NF-κB (PMID 26490297).	NF-κB<br>Therapeutic target in AML?

PSMA5	*Proteasome 20S subunit alpha 5.* The proteasome is a multicatalytic proteinase complex with a highly ordered ring-shaped 20S core structure. The core structure is composed of 4 rings of 28 non-identical subunits; 2 rings are composed of 7 alpha subunits and 2 rings are composed of 7 beta subunits. Proteasome inhibitors are now tried in the treatment of AML (PMID 31379016, 19811823), and the proteasomes are in addition important for activation of NF-κB (PMID 26490297).	NF-κB<br>Therapeutic target in AML?
HIST1H2BD	*Histone cluster 1 H2B family member D.* Expression of this gene may have a prognostic impact in cervical cancer (PMID 29184082).	Histone
TGFBR2	*Transforming growth factor beta receptor 2.* This transmembrane protein has a protein kinase domain, forms a heterodimeric complex with TGF-beta receptor type-1, and binds TGF-beta. This receptor/ligand complex phosphorylates proteins, which then enter the nucleus and regulate the transcription of genes related to regulation of cell proliferation and cell cycle progression. TGF is a regulator of the proliferation of primary human AML cells, but the final effect depends both on the biological context and patient heterogeneity (PMID 8324749, 29140182).	Transcription<br>Growth regulation in AML
NETWORK 2 (mitochondrial proteins). *This network consists of eight mitochondrial proteins; seven of them being mitochondrial ribosomal proteins and six of these proteins being a part of the larger 39S subunit. None of these individual genes have been associated with leukemogenesis or chemoresistance in human AML, but mitochondria are regarded as important both for leukemogenesis and chemosensitivity in human AML and the mitochondrial targeting is regarded as a possible therapeutic strategy in AML (PMID 25640960, 31593878, 32512867, 32318340).*		
MRPL17	*Mitochondrial ribosomal protein L17.* Mitochondrial ribosomal proteins are encoded by nuclear genes and help in protein synthesis within the mitochondrion. Mitochondrial ribosomes (mitoribosomes) consist of a small 28S subunit and a large 39S subunit. This gene encodes a 39S subunit protein.	Mitochondria<br>Ribosome
MRPL18	*Mitochondrial ribosomal protein L18.* This nuclear gene encodes a protein component of the larger 39S subunit of mitochondrial ribosome. This protein may also aid in the import of nuclear-encoded 5S rRNA into mitochondria.	Mitochondria<br>Ribosome
MRPL20	*Mitochondrial ribosomal protein L20.* This gene encodes a 39S subunit protein of the mitochondrial ribosome.	Mitochondria<br>Ribosome
MRPL22	*Mitochondrial ribosomal protein L22.* This gene encodes a 39S subunit protein of the mitochondrial ribosome; it belongs to the L22 ribosomal protein family.	Mitochondria<br>Ribosome
MRPL35	*Mitochondrial ribosomal protein L35.* This gene encodes a 39S subunit protein of the mitochondrial ribosome. Sequence analysis identified three transcript variants.	Mitochondria<br>Ribosome

MRPL50	*Mitochondrial ribosomal protein L50.* This gene encodes a putative 39S subunit protein of the mitochondrial ribosome that belongs to the L47P ribosomal protein family.	Mitochondria<br>Ribosome
NDUFA12	*NADH: ubiquinone oxidoreductase subunit A12.* This gene encodes a protein which is part of mitochondrial complex 1, i.e. a part of the oxidative phosphorylation system. Complex 1 transfers electrons to ubiquinone from NADH which establishes a proton gradient for the generation of ATP.	Mitochondria<br>Energy metabolism
MALSU1	*Mitochondrial assembly of ribosomal large subunit 1.*	Mitochondria<br>Ribosome
NETWORK 3 (spliceosomal proteins/RNA). *The importance of the spliceosome in leukemogenesis is illustrated by the detection of mutations in genes encoding proteins that are involved in RNA binding and the function of the spliceosome (PMID 27276561, 30333627). Recent studies also suggested that therapeutic targeting of RNA binding proteins can be a new therapeutic strategy in human AML, especially in patients with spliceosomal mutations (PMID 30799057, 31408619, 31578525).*		
SNRPD3	*Small nuclear ribonucleoprotein D3 polypeptide.* This protein is a core component of the spliceosome, which is a nuclear ribonucleoprotein complex that functions in pre-mRNA splicing.	Spliceosome
SNRPF	*Small nuclear ribonucleoprotein polypeptide F.*	Splicing?
SNRPG	*Small nuclear ribonucleoprotein polypeptide G.* The protein encoded by this gene is a component of the U1, U2, U4, and U5 small nuclear ribonucleoprotein complexes, precursors of the spliceosome. The encoded protein may also be a part of the U7 small nuclear ribonucleoprotein complex, which participates in the processing of the 3' end of histone transcripts.	RNA<br>Splicing/spliceosome<br>Histone
SNRPD2	*Small nuclear ribonucleoprotein D2 polypeptide.* This protein belongs to the small nuclear ribonucleoprotein core protein family. It is required for pre-mRNA splicing and small nuclear ribonucleoprotein biogenesis.	RNA<br>Splicing
SNRPE	*Small nuclear ribonucleoprotein polypeptide E.* The protein is a core component of U small nuclear ribonucleoproteins, which are key components of the pre-mRNA processing spliceosome. It plays a role in the 3' end processing of histone transcripts.	mRNA<br>Spliceosome<br>Histone
IMP3	*IMP U3 small nucleolar ribonucleoprotein 3*. This protein localizes to the nucleoli and interacts with the U3 snoRNP complex. An antiapoptotic effect with increased chemoresistance of human AML cell lines has been described; the mechanism was stabilization of and transfer of Cox-2 mRNA from the nucleus to the cytoplasm (PMID 26342105).	Nucleolus<br>RNA binding

RPS27L	*Ribosomal protein S27 like.* This protein shares 96% amino acid similarity with ribosomal protein S27, which suggests the encoded protein to be a component of the 40S ribosomal subunit.	Ribosome
NUP62	*Nucleoporin 62.* The expression of nuclear envelope proteins may have an independent prognostic impact in human AML, including the expression of nucleoporin 62 together with TMEM18, NPM35 NPM54, NPM88 and NPM133 (PMID 30199869).	Nuclear envelope<br>AML
NETWORK 4 (interferon-induced proteins). *Previous studies have shown that interferons (IFN) have antileukemic effects, but the overall results from clinical studies are modest although several clinical studies suggest a clinically relevant antileukemic effect for a subset of patients. Primary AML cells express receptors for interferons (PMID 21274002). In our opinion it is uncertain whether effects of NF-κB inhibition on INF-induced gene expression has any clinical relevance; the effects on OLR1 and especially SAMD9 may be more important. Finally, the modest effects of clinical IFN therapy may at least partly be explained by the relatively low systemic IFN levels reached after IFN therapy. Thus, even though IFN therapy has only modest antileukemic effects this does not exclude that IFN-induced proteins are involved in leukemogenesis and/or chemosensitivity.*		
IFIT1	*Interferon induced protein with tetratricopeptide repeats 1.* This protein is induced by interferon and can inhibit translational initiation.	Interferon<br>Translation
IFIT2	*Interferon induced protein with tetratricopeptide repeats 2.*	Interferon
IFIT3	*Interferon induced protein with tetratricopeptide repeats 3*	Interferon
IFIH1	*Interferon induced with helicase C domain 1.* RNA helicases are implicated in a number of cellular processes involving alteration of RNA secondary structure such as translation initiation, nuclear and mitochondrial splicing, and ribosome and spliceosome assembly. This DEAD box protein is upregulated in response to treatment with beta-interferon and protein kinase C-activation.	RNA modulation
OAS2	*2′-5′-oligoadenylate synthetase 2.* The protein is induced by interferons and uses adenosine triphosphate in 2'-specific nucleotidyl transfer reactions to synthesize 2',5'-oligoadenylates (2-5As). These molecules activate latent RNase L. The gene seem to have altered expression and may contribute to altered apoptotic regulation in the preleukemic Fanconi anemia (PMID 23845778).	Interferon<br>Fanconi anemia
SAMD9	*Sterile alpha motif domain containing 9.* This cytoplasmic protein may play a role in regulating cell proliferation and apoptosis. Mutations in this gene predisposes to development of myeloid malignancies (PMID 29535429).	Preleukemic
OLR1	*Oxidized low density lipoprotein receptor 1.* The expression of this receptor is regulated through the cyclic AMP signaling pathway and may be involved in the regulation of Fas-induced apoptosis.	Apoptosis

MX1	*MX dynamin like GTPase 1.* This guanosine triphosphate (GTP)-metabolizing protein is induced by type I and type II interferons.	Interferon

Table S3. Protein interaction networks modulated by NF-κB inhibition in human AML; a summary of interacting genes with showing increased mRNA expression in primary human AML in response to NF-κB inhibition. The table is based on the Gene database and selected references included in the PubMed database.

**PROTEIN**	**COMMENT**	**KEY WORDS**
KLF10	*Kruppel like factor 10.* This transcription factor has a C2H2-type zinc finger domains and is an effector of transforming growth factor beta signaling.	Transcription
ATF3	*Activating transcription factor 3.* This gene encodes a member of the mammalian activation transcription factor/cAMP responsive element-binding (CREB) family of transcription factors.	Transcription
NR4A2	*Nuclear receptor subfamily 4, group A, member 2.* This nuclear receptor functions as a transcriptional regulator that activates gene expression in a ligand-independent manner (PMID 22583411, 21205929).	Transcription
FOSB	*FBJ osteosarcoma oncogene B.* The protein participates in transcriptional regulation (PMID 16199154, 28454270). The gene is overexpressed in AML cells with MLL translocations (PMID 22427200), and increased levels may be a marker for susceptibility to valproic acid (PMID 19036090).	Transcription<br>Chemosensitivity
IGFBP2	*Insulin like growth factor binding protein 2.* The protein binds insulin-like growth factors I and II (IGF-I and IGF-II). It can be secreted into the bloodstream, where it binds IGF-I and IGF-II, or it can remain intracellular and interact with many different ligands. IGF is a growth factor for primary AML cells for a subset of patients (PMID 10089897).	Proliferation
MFGE8	*Milk fat globule-EGF factor 8 protein.*	-
ALB	*Albumin.* This protein enhances *in vitro* culture of primary human AML cells (PMID 3162753). Albumin was supplied in the medium in the present study. The albumin-derived EPI-X4 peptide is an endogenous inhibitor of the CXCR4 chemokine receptor and may thereby influence AML cell proliferation (PMID 32273755).	Proliferation
EIF4A1	*Eukaryotic translation initiation factor 4A1.* Normally expressed in the bone marrow.	Protein synthesis
RPLP1	*Ribosomal protein lateral stalk subunit P1.* This ribosomal phosphoprotein is a component of the 60S ribosomal subunit and plays an important role in the elongation step of protein synthesis. The protein is located in the cytoplasm.	Protein synthesis

RPLP14	*Ribosomal protein L14.* This ribosomal protein is a component of the 60S subunit and is located in the cytoplasm.	Protein synthesis
RPS11	*Ribosomal protein S11.* This member of the S17P family of ribosomal proteins is a component of the 40S subunit. The gene is co-transcribed with the small nucleolar RNA gene U35B.	Protein synthesis

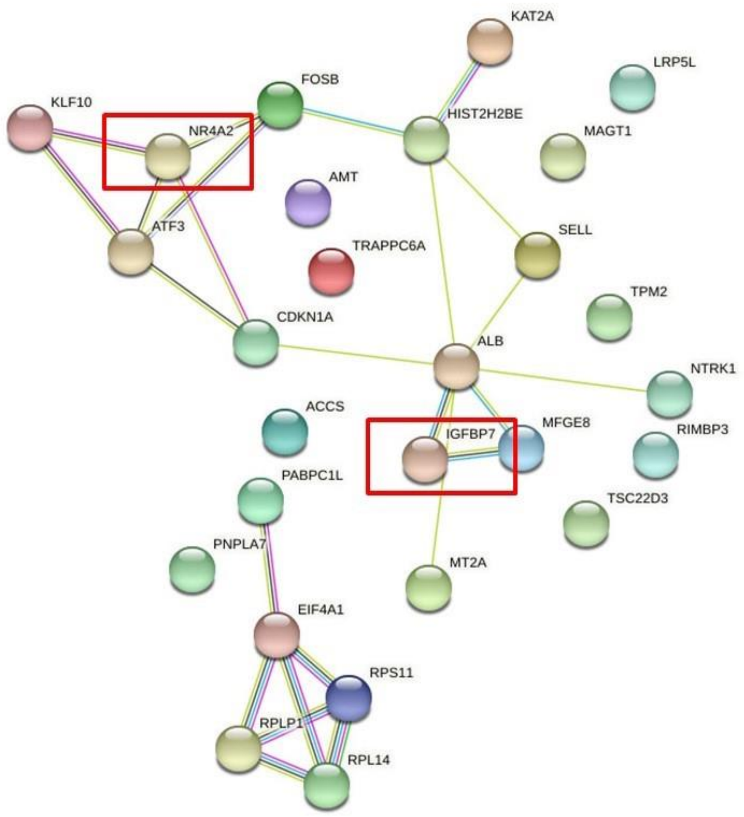

Figure S1. Network analysis of genes showing upregulated expression after NFκB inhibition. The network analysis was based on the function of the proteins encoded by the significantly upregulated genes after NF-κB inhibition. By using the STRING database we were able to identify three minor functional networks. Each node represents a single, protein-coding gene, and the number of connections indicates specific and functionally relevant interactions. Proteins known to be involved in leukemogenesis (i.e. IGFBP7 and NR4A2), are marked in the figure. The main characteristics of the encoded proteins from the three networks (KLF10-NR4A2-ATF3-FOSB-CDKN1A; ALB-IGFBP7-MFGE8; EIF4A1-RPLP1-RPS11-RPL14) are summarized in Table S3.
